# Regulation of c-Raf Stability through the CTLH Complex

**DOI:** 10.3390/ijms20040934

**Published:** 2019-02-21

**Authors:** Christina J. McTavish, Wesley Bérubé-Janzen, Xu Wang, Matthew E. R. Maitland, Louisa M. Salemi, David A. Hess, Caroline Schild-Poulter

**Affiliations:** 1 Robarts Research Institute, Schulich School of Medicine & Dentistry, The University of Western Ontario, London, ON N6A 5B7, Canada; christina.mctavish@gmail.com (C.J.M.); wesleyjanzen@hotmail.com (W.B.-J.); xwang287@uwo.ca (X.W.); mmaitla2@uwo.ca (M.E.R.M.); louisa.salemi@gmail.com (L.M.S.); dhess@robarts.ca (D.A.H.); 2 Department of Biochemistry, Schulich School of Medicine & Dentistry, The University of Western Ontario, London, ON N6A 5C1, Canada; 3 Department of Physiology and Pharmacology, Schulich School of Medicine & Dentistry, The University of Western Ontario, London, ON N6A 5C1, Canada

**Keywords:** c-Raf, CTLH complex, RanBPM, RMND5A, ubiquitination, cancer, ERK pathway

## Abstract

c-Raf is a central component of the extracellular signal-regulated kinase (ERK) pathway which is implicated in the development of many cancer types. RanBPM (Ran-Binding Protein M) was previously shown to inhibit c-Raf expression, but how this is achieved remains unclear. RanBPM is part of a recently identified E3 ubiquitin ligase complex, the CTLH (C-terminal to LisH) complex. Here, we show that the CTLH complex regulates c-Raf expression through a control of its degradation. Several domains of RanBPM were found necessary to regulate c-Raf levels, but only the C-terminal CRA (CT11-RanBPM) domain showed direct interaction with c-Raf. c-Raf ubiquitination and degradation is promoted by the CTLH complex. Furthermore, A-Raf and B-Raf protein levels are also regulated by the CTLH complex, indicating a common regulation of Raf family members. Finally, depletion of CTLH subunits RMND5A (required for meiotic nuclear division 5A) and RanBPM resulted in enhanced proliferation and loss of RanBPM promoted tumour growth in a mouse model. This study uncovers a new mode of control of c-Raf expression through regulation of its degradation by the CTLH complex. These findings also uncover a novel target of the CTLH complex, and suggest that the CTLH complex has activities that suppress cell transformation and tumour formation.

## 1. Introduction

Hyperactivation of the extracellular signal-regulated kinase (ERK) signaling pathway occurs in up to one third of human cancers of various origins and promotes cell proliferation, survival and transformation through activation of signaling pathways targeting various cytoplasmic and nuclear targets [1,2,3]. Central to these pathways are the Raf kinases, of which three isoforms exist in mammals, A-Raf, B-Raf and c-Raf which share a common structure [3]. Each isoform consists of three conserved regions (CR), each possessing their own distinct function necessary to the activity and regulation of the Raf kinases. CR1 contains the Ras binding domain necessary for Ras binding and subsequent attachment to the plasma membrane for activation. CR2 contains activating and inhibitory phosphorylation sites regulating Ras binding and Raf activation, while CR3 contains the kinase domain, which is activated upon phosphorylation of the activating segment contained in the CR3 [4,5,6], reviewed in [2]. While B-Raf has the highest occurrence of mutations in human cancers and therefore appears to have a dominant role in the ERK signaling pathway, c-Raf, also known as Raf-1, has been the most extensively studied and thus is the best characterized Raf kinase [2]. c-Raf activation is tightly regulated through a complex regulatory process involving phosphorylation/dephosphorylation events, translocation to the membrane, and subsequent homo- or heterodimerization [2]. During the inactive state, c-Raf is held in a closed conformation by the N-terminal regulatory region folding over the C-terminal catalytic domain, with the 14-3-3 dimer stabilizing the conformation by binding phosphorylated S259 (pS259) of the N-terminal, and pS621 of the C-terminal [2]. Activation is initiated by pS259 dephosphorylation, releasing 14-3-3 from the N-terminal and revealing the Ras binding sites allowing for Ras binding and recruitment to the plasma membrane [5,7]. The activation segment of CR3 is then phosphorylated, specifically at S338, to achieve full kinase activation with Raf homo- or heterodimerization, leading to its subsequent interaction with MEK [8,9]. Dimerization is required for Raf activation. Heterodimerization of c-Raf and B-Raf was demonstrated to occur following mitogen activation, while A-Raf weakly dimerizes with B-Raf [8,10]. In addition, the interaction between Heat Shock Protein 90 (Hsp90) and c-Raf is essential to c-Raf stability and its activity as a signal transducer within the ERK signaling pathway [11,12]. c-Raf stability is also regulated through ubiquitination by CHIP (C-terminus of constitutive heat shock protein (Hsc) 70-interacting protein), a highly conserved E3 ubiquitin ligase, which associates with the molecular chaperone proteins Hsc70–Hsp70 and Hsp90 causing client proteins to be ubiquitinated and subsequently degraded via the proteasome [13,14]. X-linked inhibitor of apoptosis proteins (XIAP), another E3 ubiquitin ligase, has also been found to interfere with c-Raf stability, promoting ubiquitination through Hsp90-mediated CHIP, independent of its own E3 ligase activity [15].

Interestingly, incidences of CHIP-independent modes of ubiquitination of c-Raf have been documented. For successful activation of C-Raf, S621 must be autophosphorylated in order to allow for correct folding and stability, as pS621 is necessary to bind 14-3-3 to its C-terminal [16]. Without the phosphorylation of S621, c-Raf is effectively kinase-dead and is degraded by the proteasome [17]. However, degradation is not exclusively regulated by CHIP, as siRNA knockdown of CHIP did not yield altered levels of kinase-dead c-Raf [17]. Treatment with the oxidative glucose metabolite methylglyoxal and abolishing extracellular adhesion has been shown to cause degradation of c-Raf through the ubiquitin-proteasome system [18,19]. However, the E3 ubiquitin ligase was not identified in either case.

A lesser-known regulator of c-Raf stability is the protein RanBPM (Ran-Binding Protein M), which was initially identified to bind the c-Raf kinase domain in a yeast two-hybrid analysis [20]. Subsequently, our studies showed that RanBPM and c-Raf form a complex and that RanBPM downregulates c-Raf at the protein level [21]. RanBPM also had a repressive effect on ERK phosphorylation, suggesting that, through its effect on c-Raf, RanBPM is an inhibitor of the ERK pathway [21]. However, the mechanism by which RanBPM downregulates c-Raf remains unknown. RanBPM has been previously been implicated in the regulation of several cancer pathways and has been suggested to have tumour suppressive functions [22,23]. While initially studied in isolation, it has now become evident that RanBPM is part of a large complex called the C-terminal to LisH (CTLH) complex that has E3 ligase activity [23,24]. However, the targets and activities of the CTLH complex are still largely unknown. In this study, we show that downregulation or knockout of CTLH complex subunits RanBPM and RMND5A (Required for Meiotic Nuclear Division 5A) lead to increased cell proliferation and that RanBPM downregulation promotes tumour formation in a mouse tumour model. We show that RanBPM binds c-Raf directly and that this interaction is dependent on the RanBPM C-terminal CRA (CT11-RanBPM) domain. The stability of all three Raf kinases (A-Raf, B-Raf and c-Raf) was found to be dependent on CTLH complex member RMND5A expression and c-Raf ubiquitination is regulated in a CTLH complex-dependent manner. Overall, this study uncovers a novel regulation of c-Raf by the CTLH complex that may contribute to the tumour-suppressive activity of this novel E3 ligase complex.

## 2. Results

### 2.1. CTLH Complex Members Regulate c-Raf Levels and Cell Growth

**Figure 1:**
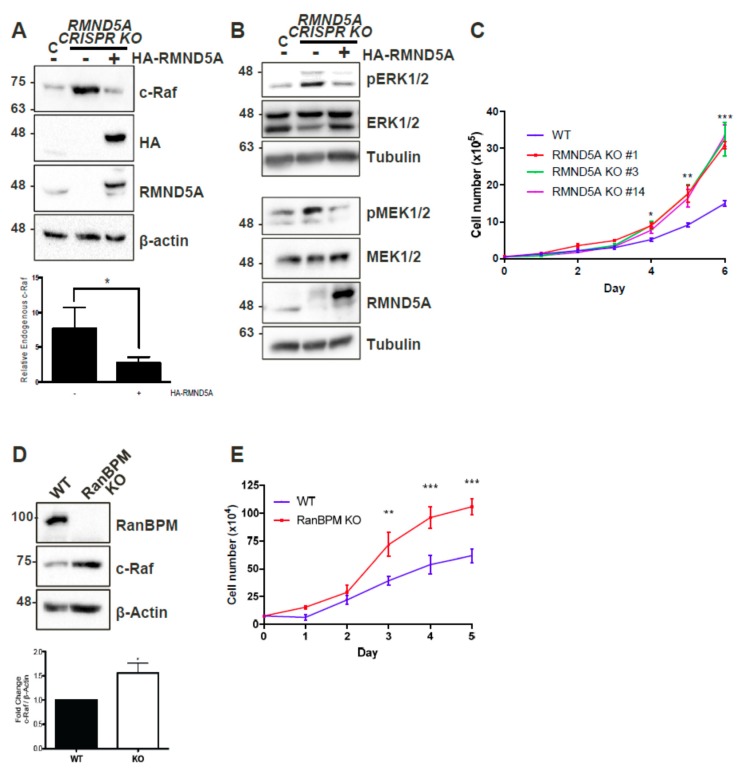
CTLH complex members RMND5A and RanBPM regulate c-Raf levels and cell proliferation. (**A**) RMND5A regulates endogenous c-Raf protein levels. Whole cell extracts from wild-type (WT) HEK293 cells and CRISPR KO RMND5A HEK293 cells untransfected (−) or transfected with pCGN-HA-RMND5A (+) were prepared and analyzed by Western blot. The top shows a representative analysis using c-Raf, HA (hemagglutinin), RMND5A, and β-actin antibodies to detect endogenous c-Raf, exogenous HA-RMND5A, endogenous RMND5A, and β-actin, respectively. Below, relative endogenous c-Raf protein levels were quantified by normalizing c-Raf to β-actin, and comparing values to wild-type HEK293 when set to a value of 1. Quantifications are shown with error bars indicating SD. *p* < 0.05 (*). (**B**) RMND5A regulates ERK signaling. Whole cell extracts from WT HEK293 cells and CRISPR KO RMND5A HEK293 cells were analyzed by Western blot for ERK and MEK phosphorylation. The same extracts were run on two different gels and equal loading was assessed for both analyses using total ERK and total MEK and a tubulin antibody. (**C**) RMND5A knockout HEK293 cells show increased proliferation. Growth rates for HEK293 control (WT, blue) and three different RMND5A CRISPR KO cell lines (clones #1, red, 3, green and 14, purple) were assessed for six days. Data represents average cell number from at least three experiments with error bars indicating SEM. *p* < 0.05 (*), *p* < 0.01 (**), *p* < 0.001 (***); (**D**) c-Raf expression is increased in primary RanBPM knockout mouse embryonic fibroblasts (MEFs). MEFs were isolated from RanBPM WT, and knockout (KO) embryos at D13.5. In the top, whole cell extracts were analyzed by Western blot with antibodies to RanBPM, c-Raf and β-actin. Below, quantification of relative amounts of c-Raf normalized to β-actin. Results are averaged from 13 paired MEFs samples from five different sets of embryos with error bars indicating SEM. *p* < 0.05 (*); (**E**) RanBPM knockout MEFs proliferate faster than WT MEFs. Growth rates for primary wildtype (WT, blue) and RanBPM knockout (KO, red) MEFs were assessed for five days. Data represents average cell number from three independent experiments performed in triplicate. Error bars represent SEM. *p* < 0.01 (**), *p* < 0.001 (***).

We previously found that RanBPM forms a complex with c-Raf and that RanBPM downregulation results in increased c-Raf expression [21]. To determine whether this effect was mediated through the CTLH complex, as opposed to a regulation by RanBPM alone, we tested c-Raf expression in cells lacking RMND5A, a CTLH complex member containing a conserved RING domain [25]. Western blot analysis of RMND5A CRISPR knockout HEK293 cell extracts showed over sixfold increase of endogenous c-Raf protein levels compared to wild-type HEK-293 (Figure 1A), which were decreased by nearly fivefold upon re-expression of RMND5A. These results demonstrate that endogenous c-Raf protein levels are affected by RMND5A expression, and therefore imply that the CTLH complex plays a role in the regulation of endogenous c-Raf protein levels.

As RanBPM downregulation was previously reported to result in increased cellular proliferation [21,26], we evaluated whether the loss of RMND5A could also confer similar properties. Comparison of growth curves of wild-type (WT) and three different RMND5A CRISPR knockout HEK293 clonal derivatives showed that control cells slowed down after four days, whereas cells lacking RMND5A proliferated markedly faster starting at day 3 (Figure 1C). We also determined that, similar to the loss of RanBPM that we previously showed induced MEK and ERK phosphorylation [21], the knockout of RMND5A resulted in increased MEK and ERK phosphorylation (Figure 1B). Interestingly, we found that primary mouse embryonic fibroblasts (MEFs) isolated from RanBPM KO mice also displayed increased c-Raf expression and increased proliferation (Figure 1D,E), suggesting that the consequences of the loss of RanBPM/CTLH complex are not restricted to immortalized cells.

### 2.2. RanBPM Expression Prevents Tumour Formation in Mouse Models

Our observations that RanBPM downregulation promotes c-Raf expression and ERK activation [21] suggested that loss of RanBPM function could promote tumour formation in vivo. Moreover, downregulation of RanBPM in Hela and HCT116 cells causes extensive changes in the expression of several genes implicated in oncogenesis [27]. In particular, overexpression of RON (Recepteur d’origine nantais) kinase, L1 cell adhesion molecule (L1CAM), ELF3 (E74-like factor 3), transglutaminase 2 (TG2) (all increased in RanBPM shRNA cells [27]) have all been reported in various tumour types and were shown to be directly implicated in cancer development [28,29,30,31]. Thus, loss of RanBPM affects several pathways which collectively promote many aspects of tumorigenesis.

**Figure 2:**
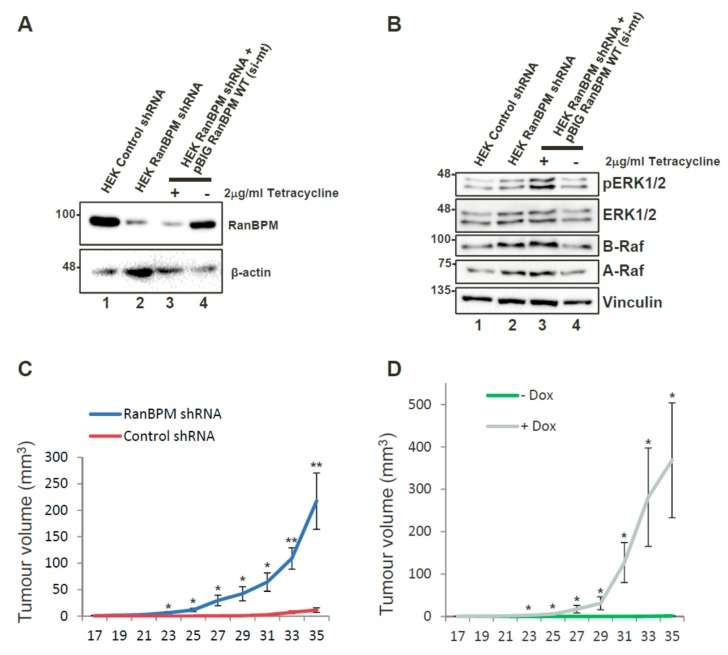
Downregulation of RanBPM promotes tumour formation in NOD/SCID/gamma mice. (**A**) re-expression of RanBPM in HEK293 cells via Tet-off pBIG expression vector. HEK293 pool of cells stably expressing RanBPM shRNA were transfected with pBIG-RanBPM WT (si-mt) and maintained in media with 2 μg/mL Tetracycline and 250 μg/mL hygromycin to select for integration of the pBIG vector. Following selection, cells were either maintained (+) in Tetracyclin-containing media, or cultured in absence of Tetracyclin (−) for 24 h to allow induction of RanBPM. Tetracyclin removal leads to re-expression of RanBPM (lane 4); (**B**) ERK pathway activation in RanBPM shRNA cells. Samples shown in (**A**) were analyzed for ERK phosphorylation and A-Raf and B-Raf expression. The Western blot was analyzed with the indicated antibodies; (**C**) injections with HEK293 control and RanBPM shRNA pools were injected subcutaneously in the flank of 6–8 weeks old NOD/SCID/gamma. Tumour measurements were taken twice per week and a digital caliper was used to measure Length × Width × Depth of the tumour upon excision in order to calculate volume. *n* = 7, error bars represent SEM; (**D**) injections with HEK293 RanBPM shRNA pool of cells stably re-expressing RanBPM via pBIG Tet-off expression system (see **C**, lanes 3,4). Mice were fed chow containing Dox (purple line) or regular chow (green line). *n* = 6, error bars represent SEM. *p* < 0.05 (*), *p* < 0.01 (**). Error bars are included for all data points but may not be visible when smaller than line size.

We tested whether RanBPM downregulation could promote tumour formation in a xenograft model. For this assay, we generated a pool of early passage HEK293 cells stably expressing RanBPM shRNA or control shRNA (Figure 2A). HEK293 cells are immortalized with Adenovirus 5 E1A expression but exhibit weak tumorigenicity [32]. RanBPM shRNA HEK293 injected into NOD/SCID/gamma (NSG) mice caused a marked and statistically significant increase in tumour volume over control cells (Figure 2C). Tumours also appeared earlier (day 17, versus day 31) and were on average twentyfold larger (220 mm^3^ versus 11 mm^3^) than those observed with control cells at day 35. These results suggest that RanBPM downregulation can enhance tumorigenic properties of HEK293 cells in this model. To demonstrate that these effects were not due to off-target effects of the RanBPM shRNA, we generated HEK293 RanBPM shRNA cells with stably integrated Tetracyclin (Tet)-off vector pBIG-RanBPM in which RanBPM can be re-expressed upon removal of Tet/doxycycline (Dox) (Figure 2A, lanes 3,4). We verified that re-expression of RanBPM reduced ERK phosphorylation to wild-type levels (Figure 2B). Interestingly, RanBPM shRNA cells also showed an upregulation of A-Raf and B-Raf protein levels, which specifically decreased upon re-expression of RanBPM, suggesting that ERK pathway activation could result from all three Raf kinases activation in these cells. pBIG RanBPM shRNA cells were injected into NSG mice. Mice fed with Dox-containing chow developed tumours, whereas those fed with regular chow did not, demonstrating that RanBPM re-expression effectively prevents tumour formation (Figure 2D).

### 2.3. Both Endogenous RanBPM and MAEA Form a Complex with c-Raf In Situ

Previous co-immunoprecipitation and pull-down experiments in our laboratory have demonstrated that RanBPM and c-Raf exist together in a complex [21]. Since this was shown using ectopically expressed protein constructs, we sought to confirm that the complex occurs with the endogenous proteins in vivo. The in situ Proximity Ligation Assay (PLA), which allows visualization of protein–protein interactions in cells using fluorescence microscopy [33], was thus used to visualize the interaction between endogenous CTLH complex members MAEA (Macrophage erythroblast attacher) and RanBPM and c-Raf in HeLa cells.

**Figure 3:**
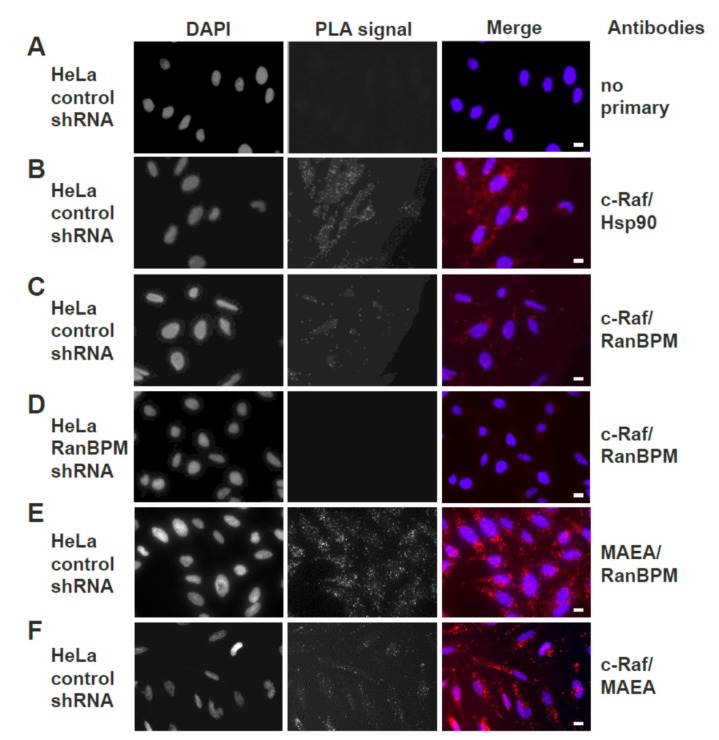
Endogenous RanBPM and c-Raf interaction in HeLa cells using PLA. Duolink II proximity ligation assay (PLA) was performed in: (**A**) control shRNA HeLa cells, without the addition of primary antibodies (negative control); (**B**) control shRNA HeLa cells, with Hsp90 and c-Raf primary antibodies (positive control); (**C**) control shRNA HeLa cells, using c-Raf and RanBPM primary antibodies; (**D**). HeLa RanBPM shRNA cells, with c-Raf and RanBPM primary antibodies (negative control); (**E**) control shRNA HeLa cells, using MAEA (Macrophage erythroblast attacher) and RanBPM primary antibodies (positive control). (**F**) Control shRNA HeLa cells, using c-Raf and MAEA primary antibodies. The DAPI filter was used to visualize the nuclei, while the Cyanine 3 (Cy3) filter was used to visualize the PLA dots representing protein–protein interactions. Representative images from one of three independent experiments are shown. Scale bars, 10 μm.

Stable HeLa control or RanBPM shRNA (clone 2–7), previously generated in our laboratory [34], were used in this experiment. As a control, HeLa cells were incubated without primary antibodies (Figure 3A). Given that c-Raf is known to interact with Hsp90 [35], a positive control was included where HeLa control cells were incubated with antibodies against c-Raf and Hsp90. This did expectedly produce fluorescent dots representing interactions (Figure 3B). In HeLa control cells in which antibodies against c-Raf and RanBPM were included for the assay, fluorescent dots were observed, confirming that the two proteins are found together in a complex in vivo (Figure 3C). To confirm the specificity of the RanBPM primary antibody, another control was included where HeLa RanBPM shRNA cells were incubated with primary antibodies against RanBPM and c-Raf (Figure 3D). As expected, fluorescent dots representing interactions were not observed in absence of RanBPM. To confirm that c-Raf is interacting with the CTLH complex, we repeated the experiment with CTLH complex member MAEA. A positive control using primary antibodies against MAEA and RanBPM presented numerous dots, consistent with these two proteins being present in a complex (Figure 3E). MAEA and c-Raf antibodies also yielded fluorescent dots (although in noticeably fewer numbers than the MAEA and RanBPM) indicative of the close proximity of the two proteins (Figure 3F), suggesting that endogenous c-Raf associates with the CTLH complex as a whole in vivo.

### 2.4. The N-Terminus, CRA and LisH/CTLH Domains of RanBPM Are Required for c-Raf Downregulation

**Figure 4:**
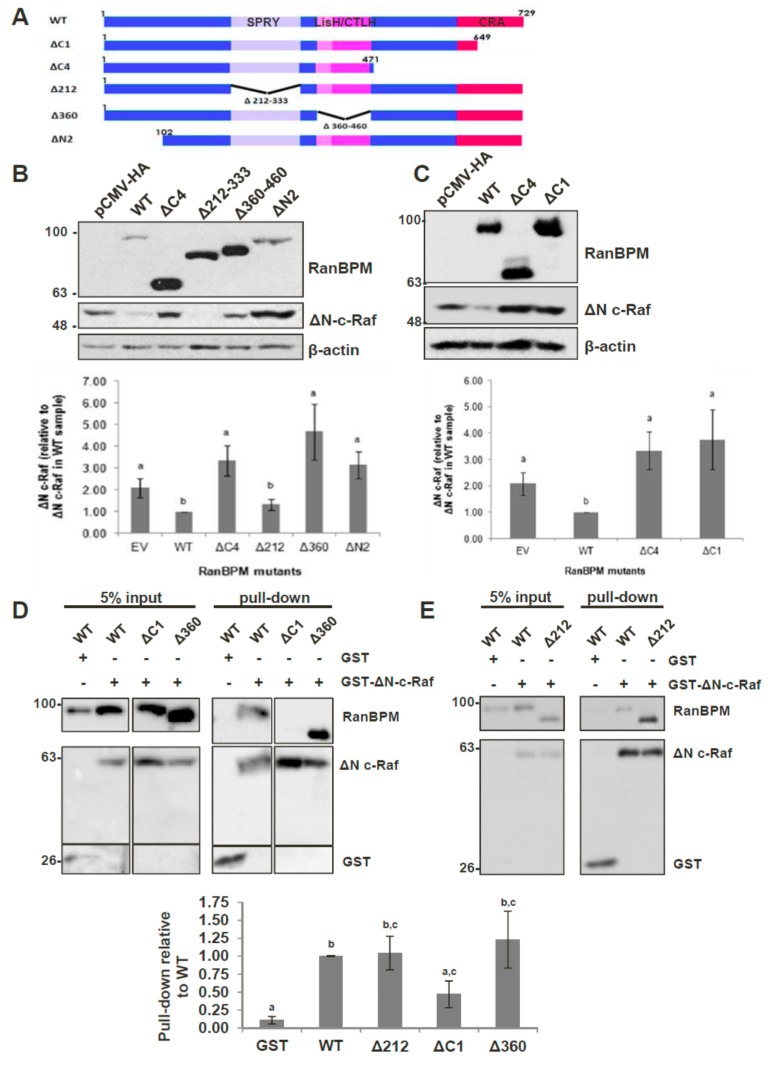
Analysis of RanBPM domains that control C-Raf stability. (**A**) schematic representation of RanBPM mutants. (**B**) and (**C**) Western blot analyses of HeLa RanBPM shRNA cells transfected with pEBG-GST-ΔN-c-Raf and either pCMV-HA (empty vector), pCMV-HA-WT-RanBPM or pCMV-HA RanBPM mutant constructs as indicated. c-Raf and HA antibodies were used to detect the levels of ΔN-c-Raf and RanBPM, respectively. β-actin was used as a loading control. A representative Western blot is shown (top) and quantifications of c-Raf levels are shown (bottom graph) with error bars indicating SEM (*n* = 5). Deletion of RanBPM C-terminal domain (ΔC1) impairs RanBPM interaction with GST-ΔN-c-Raf. (**D**) and (**E**) GST-Pull-down assays. HeLa RanBPM shRNA cells were transfected with pEBG-ΔN-c-Raf and either pCMV-HA (empty vector), pCMV-HA-WT-RanBPM or pCMV-HA RanBPM mutant constructs. ΔN-c-Raf was pulled down through binding to glutathione-sepharose beads and interaction of RanBPM WT and mutants with GST-ΔN-c-Raf assessed by Western blot with an HA antibody. Below: Quantifications were performed by normalizing RanBPM mutant levels to pulled-down GST or GST-ΔN-c-Raf and statistical analyses were performed (*n* = 4–7, SEM shown). Different letters are statistically different (*p* < 0.05).

Given that RanBPM contains a number of conserved domains, we sought to determine which regions of RanBPM were necessary for downregulation of c-Raf. We used a series of RanBPM deletion mutant constructs (Figure 4A) sub-cloned into the mammalian expression vector pCMV-HA [34,36], to test their effects on levels of c-Raf. ΔN-c-Raf, a constitutively active construct of c-Raf containing only amino acids 325–648 [9], was used instead of full-length c-Raf because RanBPM has been shown to have a greater effect on activated c-Raf [21]. Since RanBPM is able to dimerize [37], we performed the experiments in HeLa RanBPM shRNA cells to avoid dimerization between mutant RanBPM and endogenous WT RanBPM monomers. As previously reported [21], WT RanBPM was able to significantly downregulate ΔN-c-Raf compared to the levels of ΔN-c-Raf seen in the pCMV-HA control (Figure 4B). The RanBPM deletion mutant Δ212, in which the SPRY domain is deleted, was also able to downregulate ΔN-c-Raf, indicating that the SPRY domain is not required for c-Raf destabilization.

Next, we tested the effect of N-terminal deletions of RanBPM. We previously reported that ΔN2 RanBPM is expressed at lower levels than WT RanBPM [36], which made the results difficult to evaluate since lower expression levels may have accounted for the inefficiency of this mutant to regulate ΔN-c-Raf. However, even in experiments where higher levels of ΔN2 RanBPM were obtained, ΔN2 RanBPM was unable to effectively downregulate ΔN-c-Raf (Figure 4B). Therefore, ΔN2 RanBPM appeared to have lost its ability to downregulate ΔN-c-Raf, implying that the N-terminus of RanBPM is required for its effect on c-Raf.

The Δ360 and ΔC4 RanBPM mutants also did not effectively downregulate ΔN-c-Raf, as ΔN-c-Raf expression levels were significantly higher than those seen in response to WT RanBPM, and not significantly different than those seen in the pCMV-HA control (Figure 4B). This occurred despite the fact that expression of these mutants was noticeably higher than that of WT RanBPM. These results suggest that the LisH/CTLH domains and the C-terminus of RanBPM play a role in c-Raf downregulation. However, considering that the ΔC4 deletion removes a very large portion of RanBPM, we repeated the experiment using a construct harboring only a deletion of the CRA domain, namely the ΔC1 RanBPM construct. Like ΔC4, ΔC1 was unable to downregulate ΔN-c-Raf (Figure 4C). This implies that, within the C-terminus of RanBPM, it is specifically the CRA domain that is needed for c-Raf downregulation.

Altogether, these results demonstrate that the N-terminus, LisH/CTLH and CRA domains are required for c-Raf destabilization, since loss of any of these regions render RanBPM unable to effectively downregulate c-Raf.

### 2.5. The CRA Domain of RanBPM Is Required for Interaction with c-Raf

Next, we aimed to determine the RanBPM domain(s) required for interaction with c-Raf. To accomplish this, we co-transfected either GST or GST-ΔN-c-Raf with RanBPM deletion mutants in RanBPM shRNA HeLa cells and performed GST pull-down assays. Due to its poor stability, ΔN2 RanBPM was not tested as we were unable to obtain sufficient levels of the protein to detect it in this type of assay. As anticipated based on previous studies [21], GST-ΔN-c-Raf was able to successfully pull-down WT RanBPM (Figure 4D). Both Δ212 RanBPM and Δ360 RanBPM associated with ΔN-c-Raf similar to WT, indicating that that the SPRY and LisH/CTLH domains are not required for the interaction between RanBPM and c-Raf (Figure 4D,E). However, ΔC1 RanBPM was not able to effectively interact with ΔN-c-Raf, showing a twofold decrease compared to WT RanBPM (Figure 4D). Thus, the data suggest that, of the domains tested here, the CRA domain is the only one required for the interaction between RanBPM and c-Raf.

### 2.6. RanBPM Interacts Directly with c-Raf through the CRA Domain

**Figure 5:**
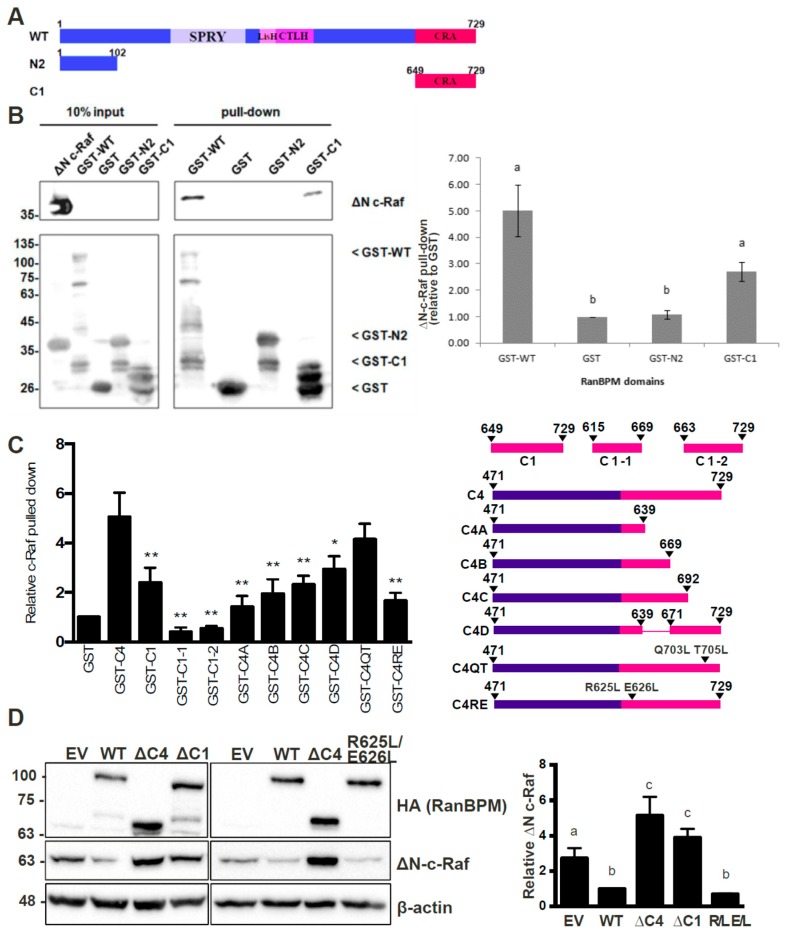
RanBPM C-terminal CRA domain directly interacts with ΔN-c-Raf and is necessary for C-Raf regulation. (**A**) diagram of WT RanBPM, N2 domain and C1 domain cloned into the bacterial expression vector pGEX-4T-1; (**B**) Left, Western blot analysis of GST pull-down assays for N c-Raf performed using GST, GST-WT-RanBPM, GST-N2-domain and GST-C1-domain *E. coli* extracts. A representative image is shown. Right, pull down assays experiments were quantified by normalizing ΔN-c-Raf levels to pulled-down GST, GST-WT-RanBPM, GST-N2 or GST-C1 and statistical analyses were performed (*n* = 6, error bar indicates SEM) with different letters indicating statistical difference (*p* < 0.05); (**C**) analysis of RanBPM CRA mutants using in vitro pull-down. Right, schematic representation of the mutants analyzed. Left, Quantifications of pull down experiments performed with the CRA mutants as described in (**B**). Relative ∆N-C-Raf protein levels were quantified by normalizing ∆N-C-Raf to the GST-fusion protein product, and comparing values to GST when set to a value of 1. Quantifications are shown with error bars indicating SD (*n* = 3–5). Statistical difference with respect to GST-C4 is indicated, * *p* < 0.05; ** *p* < 0.01; (**D**) analysis of RanBPM CRA domain mutants in mammalian cells. HeLa RanBPM shRNA cells were transfected with pEBG-GST-ΔN-c-Raf and either pCMV-HA empty vector, RanBPM WT, RanBPM-ΔC4, RanBPM-ΔC1, or RanBPM-R625L E626L, and whole cell extracts were prepared 24 h post-transfection and analyzed by Western blot. HA, c-Raf and β-actin antibodies were used to detect HA-RanBPM constructs, ∆N-c-Raf and β-actin proteins, respectively. Right, relative ∆N-c-Raf protein levels were quantified by normalizing ∆N-c-Raf to β-actin, and comparing values to HA-WT when set to a value of 1. Different letters are statistically different (*p* < 0.05). Error bars indicate SD (*n* = 4).

To confirm the interaction between the CRA domain of RanBPM and c-Raf and determine whether the interaction is direct, we performed pull-down experiments using bacterially expressed mammalian c-Raf and RanBPM. In addition to full-length RanBPM, we tested RanBPM C1 (encoding only the CRA domain) and the N2 region (Figure 5A), which was unexamined in the mammalian cell-based GST pull-down assays.

GST-RanBPM constructs were purified on glutathione beads and incubated with a crude cell lysate from *E. coli* expressing ΔN-c-Raf. Both GST-WT-RanBPM and GST-C1 were able to pull-down ΔN-c-Raf (Figure 5B), indicating that the interaction between RanBPM and c-Raf is direct and that the CRA domain is sufficient for the interaction. GST-N2 was unable to pull-down ΔN-c-Raf (Figure 5B), suggesting that the N-terminus of RanBPM is unable to directly interact with c-Raf.

### 2.7. Analysis of the RanBPM CRA Domain Interaction with c-Raf

To identify sub-domains that might mediate interaction with c-Raf, we examined structural features of the CRA domain to design partial deletions. As there is no crystal structure of RanBPM or the CRA domain currently available, a predicted tertiary structure was elucidated by the RAPTORX online server [38]. The predicted tertiary structure corroborated the previous assumption that the CRA domain has a high propensity for α-helices, with 6 α-helices spanning the entirety of the domain [23,39] (Figure S1A). Deletion mutants of the RanBPM CRA domain were created to identify sub-domains that might mediate interaction with c-Raf (Figure 5C). Deletions were guided by thepredicted tertiary structure (Figure S1B) and data shown above demonstrating that amino acids 649–729 of RanBPM, denoted C1, bound directly to ΔN-c-Raf (Figure 5B). Only a portion of the CRA domain is present in the C1 construct, namely helices IV, V and VI, and a small portion of helix III. We therefore derived two GST-fusion CRA constructs which contained amino acids 615–669 spanning helices I, II, and III, denoted C1-1, and amino acids 663–729 containing helices IV, V, and VI, denoted C1-2. GST pull down assays were normalized to the negative control, GST, and compared to the positive ∆N-c-Raf binding control, GST-C4, which contains amino acids 471–729 of RanBPM. (Figure 5C, representative experiments shown in Figure S2A). GST-C1 displayed significant interaction with ΔN-c-Raf, although quantifications showed reduced binding compared to GST-C4, by 2.5-fold. Both GST-C1-1 and GST-C1-2 appeared to lack protein stability as they bound less ΔN-c-Raf than that of the GST negative control.

To increase protein stability, CRA domain constructs were expanded to include sequences N-terminal to the CRA domain (Figure 5C). Similar to the strategy used for the two previous constructs, CRA deletion mutations were guided by the predicted tertiary structure. We derived an additional four GST-fusion CRA constructs: C4A which spans amino acids 471–639 and helices I and II; C4B contains amino acids 471–669 and helices I–III; C4C is comprised of amino acids 471–692 and helices I–IV; and finally, C4D which includes amino acids 471–639 and 671–729, and helices I–II and IV–VI. ΔN-c-Raf binding was again tested by GST pull down assay and the mutants binding to ΔN-c-Raf were quantified and compared to that of GST-C4 (Figure 5C and Figure S2B). All mutants showed decreased binding compared to C4, suggesting that each helix of the CRA domain plays a role in binding ΔN-c-Raf. As more helices were deleted from the CRA domain, less binding of ΔN-c-Raf was observed. However, two portions of the CRA domain seemed worth investigating, helices I and II, and helices V and VI, since deletion of helices I and II (C1), and helices V and VI (C4C) resulted in a sharp decrease in ΔN-c-Raf binding with the least amount of the CRA domain deleted.

Point mutations were designed to target the center of helices I and II, or helix V, in efforts to disrupt the helical structures within the CRA domain and the binding interface between CRA and ΔN-c-Raf. Site-directed mutagenesis was used to create C4 with point mutations R625L and E626L within helix I and II, denoted C4-R625L E626L, or with point mutations Q703L and T705L within helix V, denoted C4-Q703L T705L. GST-C4-R625L E626L showed a significant loss in ΔN-c-Raf binding with over threefold decrease compared to C4, whereas GST-C4-Q703L T705L did not disrupt the interaction, showing only a slight decrease in ΔN-c-Raf binding (Figure 5C and Figure S2C). These results suggest that the mutation R625L E626L made to helix I and II of the CRA domain, is sufficient to disrupt the interaction between the CRA domain and ΔN-c-Raf in vitro.

To validate the in vitro binding data, mutant RanBPM CRA domains were cloned into the mammalian expression vector pCMV, and co-transfected with pEBG-GST ΔN-c-Raf in HeLa RanBPM shRNA cells. RanBPM- ΔC1 and RanBPM- R625L E626L were chosen to investigate their effect on ΔN-c-Raf regulation. RanBPM-ΔC1 showed significant loss of ΔN-c-Raf regulation, compared to WT RanBPM, confirming our in vitro binding data (Figure 5D). However, the RanBPM R625L E626L point mutant maintained the regulation of ΔN-c-Raf protein levels to a level comparable to WT RanBPM, suggesting that the mutant could still interact with c-Raf in the cellular context. Similarly, the C4C deletion (693–729) did not affect ΔN-c-Raf regulation when transfected in Hela RanBPM shRNA cells (Figure S2D). This suggests that the interaction in vivo may be enhanced or stabilized by other domains of RanBPM or by other members of the CTLH complex.

### 2.8. c-Raf Stability Is Regulated through the Proteasome and Its Ubiquitination Is Dependent on the CTLH Complex

**Figure 6:**
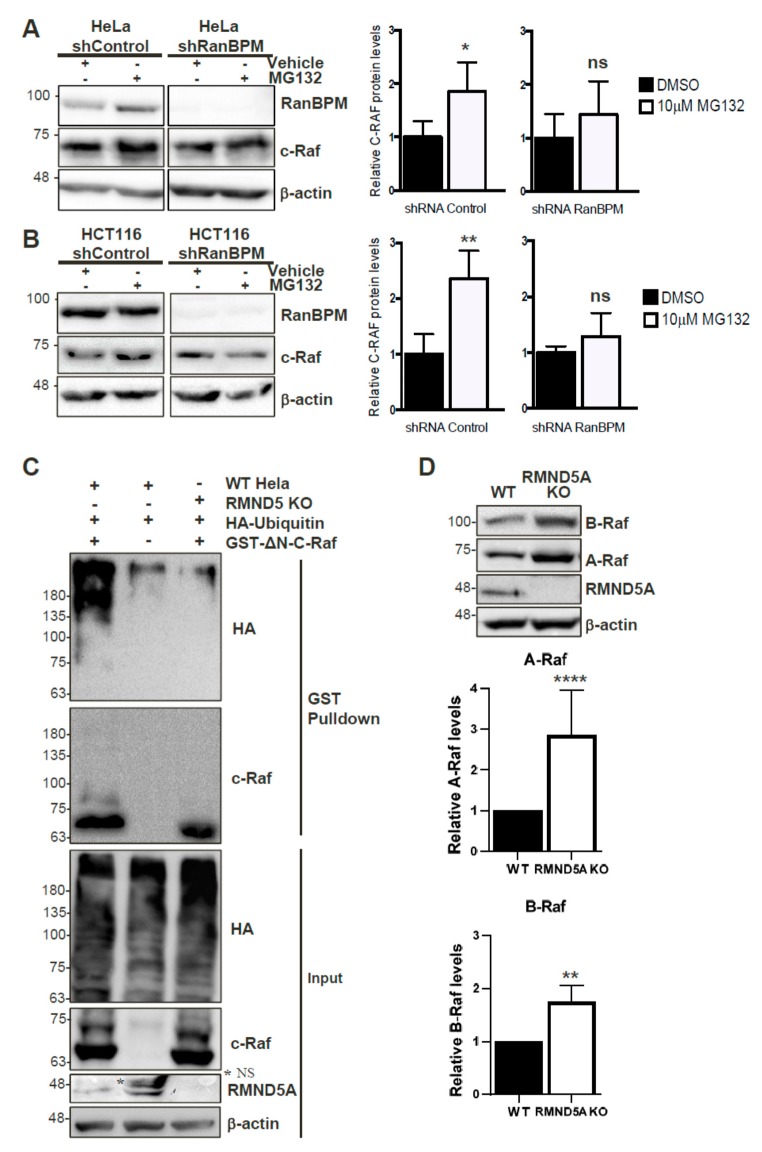
C-Raf is regulated by the proteasome through the CTLH complex. Non-targeting shRNA control and shRNA RanBPM cells were treated with 10 μM MG132 or DMSO, as vehicle, for 24 h. RIPA buffered whole cell extracts of HeLa (**A**), and HCT116 (**B**) were analyzed by Western blot with RanBPM, c-Raf and β-actin antibodies to detect RanBPM, c-Raf and β-actin proteins, respectively. c-Raf protein levels were normalized to β-actin levels. Quantifications of relative c-Raf protein levels are shown with error bars indicating SD (*n* = 4). * *p* < 0.05; ** *p* < 0.01; ns, no significance. (**C**) c-Raf is ubiquitinated in a CTLH complex-dependent manner. Hela control (WT Hela) and RMND5A KO cells were transfected with GST-ΔN-c-Raf and/or HA-Ubiquitin as indicated. GST pull-downs performed using whole cell extracts were analyzed by Western blot with the indicated antibodies. Input represent 5% of the extracts used for pull-down. The asterix (*) indicates a non-specific band (NS). (**D**) A-Raf and B-Raf expression is increased in RMND5A KO cells. Extracts from HEK293 control and RMND5A CRISPR KO cells were analyzed by Western blot with the indicated antibodies. Quantifications are shown below with error bar indicating SD, *n* = 8 for A-Raf, **** *p* < 0.0001 and *n* = 3 for B-Raf, ** *p* < 0.01.

Since the CTLH complex has E3 ubiquitin ligase activity [24], we wanted to determine whether it regulates c-Raf protein stability through the proteasomal degradation pathway. RanBPM shRNA and control shRNA HeLa, and HCT116 cells were used to assess c-Raf protein levels in conditions of proteasomal impairment in order to determine whether RanBPM loss of function would affect c-Raf degradation. All cell lines were treated with either 10 µM of the proteasome inhibitor, MG132, or with the vehicle, dimethyl sulfoxide (DMSO). HeLa and HCT116 cells expressing RanBPM shRNA showed minimal accumulation of c-Raf protein levels during proteasomal inhibition, whereas control shRNA cells exhibited a significant increase of c-Raf expression when compared to their respective vehicle-treated controls (Figure 6A,B). This suggests that the downregulation of RanBPM impairs proteasome-dependent degradation of c-Raf. To determine whether this could occur through ubiquitination of c-Raf by the CTLH complex, we co-transfected control and RMND5A KO HeLa cells with GST-ΔN-c-Raf and HA-Ubiquitin constructs to investigate the involvement of RMND5A in c-Raf ubiquitination. GST-ΔN-c-Raf ubiquitination was observed in control Hela cells but was strongly reduced in RMND5A KO cells confirming that the CTLH complex regulates c-Raf ubiquitination (Figure 6C). Taken together, our data suggests that c-Raf ubiquitination and proteasomal degradation is regulated by the CTLH complex.

### 2.9. The CTLH Complex Regulation Is Conserved for A- and B-Raf

The c-Raf region that interacts with the RanBPM/CTLH complex comprises the CR3 (Conserved Region 3) which is the most conserved region between the three Raf isoforms and includes the kinase domain [2]. Our observation that RanBPM shRNA cells display increased expression of A-Raf and B-Raf (Figure 2B) prompted us to evaluate whether RMND5A could also regulate the levels of the other two Raf kinases, A-Raf and B-Raf, which would confirm the involvement of the CTLH complex in the regulation of all three Raf kinases. Indeed, levels of both A- and B-Raf were increased in RMND5A KO cells (Figure 6D), with the most pronounced effect on A-Raf, which showed an almost threefold increase compared to nearly twofold for B-Raf. This effect was not due to an isolated clonal effect, as all RMND5A CRISPR KO clones obtained showed increased expression of the three Raf kinases compared to wild-type cells (Figure S3). This suggests that the CTLH complex regulates all three Raf kinases likely through a similar mechanism.

## 3. Discussion

**Figure 7:**
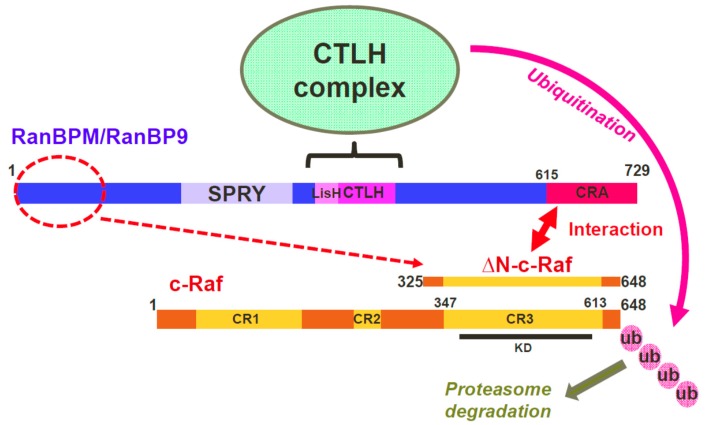
Model of regulation of c-Raf by the RanBPM/CTLH complex. Three RanBPM regions, N-terminal (1–102), LisH/CTLH (360–460) and C-terminal CRA (615–729) are necessary to regulate c-Raf expression/stability, but only the CRA domain is able to directly interact with c-Raf in vitro. Our data suggest that RanBPM interacts with c-Raf through the CRA domain and recruits c-Raf to the CTLH complex to which RanBPM is associated through its LisH/CTLH domain. The CTLH complex promotes c-Raf ubiquitination and degradation. The role of RanBPM N-terminal domain is unclear, but it may be involved in RanBPM stability and folding and potentially stabilizes c-Raf interaction (dashed line). The minimum region of c-Raf defined so far as necessary for interaction with RanBPM is ∆N-c-Raf, which is comprised of conserved region CR3 and short flanking sequences. The position of the CR1, CR2 and CR3 conserved regions are shown. The location of the c-Raf catalytic domain (KD, kinase domain) is indicated. The thick double-head arrow indicates interaction. The dashed arrow indicates a regulation of c-Raf by the RanBPM N-terminal domain. Ubiquitination of c-Raf by the CTLH complex is indicated by the pink arrow. The bracket indicates that the LiSH/CTLH domain mediates interaction with CTLH complex members.

The regulation of Raf kinases is central to the signaling activities of the ERK pathway [1,2,3]. In this study, we have identified a novel regulatory mechanism of c-Raf protein expression. The data presented here show that the CTLH complex, a yet uncharacterized E3 ubiquitin ligase complex, affects c-Raf stability and promotes its ubiquitination. One of the CTLH complex subunits, RanBPM, interacts directly with the C-terminal domain of c-Raf through its CRA domain. Preventing this interaction or interfering with CTLH subunit expression results in increased c-Raf expression and loss of its ubiquitination. Our data suggest a model whereby RanBPM is the targeting subunit necessary for c-Raf recruitment to the CTLH complex for ubiquitination (Figure 7).

Based on the results obtained from this study, we propose a mechanism by which RanBPM downregulates c-Raf through tethering c-Raf to the CTLH complex for ubiquitination. We showed that both MAEA and RanBPM co-localize with c-Raf using PLA in HeLa cells. In addition, c-Raf protein levels were affected by loss of CTLH complex members RMND5A and RanBPM. Deletion of the CRA domain of RanBPM prevented c-Raf downregulation, suggesting the RanBPM CRA domain is essential for the targeting of c-Raf by the CTLH complex. In addition, deletion of RanBPM CRA domain prevented the interaction between RanBPM and c-Raf and the RanBPM CRA domain alone was capable of interacting directly with c-Raf. This suggests that RanBPM recruits c-Raf to the CTLH complex through a direct interaction with the CRA domain. Deletion of the LisH/CTLH domains also inhibited c-Raf downregulation; however, RanBPM still retained its ability to interact with c-Raf. While the CTLH complex topology remains to be confirmed, studies of its yeast counterpart, the GID complex, have shown that RanBPM is connected to the other CTLH complex members through its LisH and CTLH domains [40]. Therefore, the RanBPM LisH/CTLH deletion mutant would still retain the ability to interact with c-Raf, but would no longer be able to connect c-Raf to the CTLH complex for ubiquitination. Deletion of the N-terminus of RanBPM also resulted in a loss of c-Raf downregulation while not interacting directly with c-Raf. RanBPM N-terminus is proline-rich and predicted to be an unstructured, flexible region which could fold over to stabilize the protein or promote interactions with other proteins [41]. The N-terminus could therefore be involved in stabilizing c-Raf interaction with the CRA domain.

We were unable to identify a distinct motif in the CRA domain that mediates c-Raf interaction. Our data suggest that each helix of the CRA domain has a role to play in c-Raf binding, with helices I, II, V and VI being most important for the interaction. Site-directed mutagenesis was used to alter the secondary structure of the CRA domain helices I and II or helix V in hopes of disrupting the interaction between the CRA domain and c-Raf. In vitro binding data suggested that the amino acid substitutions in helices I and II (R625L/E626L) were effective in disrupting this interaction. However, ectopically expressed RanBPM-R625L/E626L was unable to downregulate ∆N-c-Raf, suggesting that the point mutations did not disrupt the interaction between the CRA domain when tested in mammalian cells. Our in vitro experiments employed CRA domain constructs lacking the central and N-terminal portions of RanBPM. Therefore, the direct interaction of c-Raf with the RanBPM CRA domain may be stabilized by additional interactions with the RanBPM N-terminal domain and/or other CTLH complex members. It is worth noting that three other CTLH subunits, TWA1 and the two RING domain subunits MAEA and RMND5A, also contain CRA domains [25], thus it is possible that these subunits also help stabilize the c-Raf interaction with RanBPM.

We propose that c-Raf is targeted for degradation by the CTLH complex in a RanBPM-dependent manner, with c-Raf being tethered to the complex by RanBPM through its CRA domain. RanBPM is a promiscuous protein, reported to interact with over 75 proteins [23]. The ability of RanBPM to associate with many proteins has given rise to the notion that it functions as an adaptor or scaffolding protein and that it may function to bring substrate proteins into proximity of the complex [23,25,40].

Our data show that, in addition to c-Raf, both A- and B-Raf protein levels are regulated by the CTLH complex. The c-Raf region interacting with RanBPM (∆N-c-Raf, aa 325–648) consists of little more than the conserved region 3 (CR3) which is extremely well conserved between the three Raf kinases and contains the kinase domain [2]. RanBPM could therefore interact with either of the Raf kinases to target them for degradation, although an interaction between RanBPM and A- and B-Raf remains to be demonstrated.

Four other E3 ubiquitin ligases have previously been shown to be involved in c-Raf ubiquitination/degradation. The CHIP E3 ligase was shown to regulate c-Raf ubiquitination, however, CHIP downregulation only partially affected c-Raf degradation suggesting that alternative mechanisms exist involving (an) unidentified E3 ligase(s) [17]. The E3 ligase XIAP was shown to promote c-Raf ubiquitination, but this occurred in a RING-independent manner, suggesting that XIAP does not target c-Raf directly [15]. A recent study identified the E3 ubiquitin ligase HERC1 as a regulator of the ERK pathway through a regulation of c-Raf stability [42]. HERC1 was shown to interact with c-Raf and mediate c-Raf ubiquitination. Finally, the E3 ubiquitin ligase HUWE1, was also shown to regulate c-Raf degradation, functioning indirectly through ubiquitination of the Raf scaffold protein Shoc2 [43]. Whether the CTLH complex directly targets c-Raf or targets other components of the c-Raf regulatory complex remains to be investigated.

The functions and targets of the CTLH complex are still elusive, however several subunits of the complex, studied in isolation, have been implicated in various cellular processes and regulations [23]. RanBPM in particular has been implicated in the regulation of several signaling pathways involved in cancer development [22,23]. The basis for this study was our previous observation that RanBPM downregulation correlated with ERK phosphorylation and pathway activation [21]. Our results presented here suggest that RanBPM is the CTLH complex subunit that recruits c-Raf for degradation, therefore restricting the ERK pathway activation. This suggests a tumour-suppressive role for the CTLH complex, which is further supported by the fact that loss of CTLH subunits promotes cell proliferation and tumour growth in mice. What is unknown is how much of these effects are due to the ERK pathway repression since the CTLH complex may be regulating many other pathways. In particular, we have shown that the CTLH complex inhibits the activity of Histone Deacetylase 6 (HDAC6) [41,44], a deacetylase known to promote many aspects of cell transformation and oncogenesis [45]. Other pathways, such as the Src/Akt pathway and Transforming Growth Factor (TGF)-β signaling have been shown to be inhibited by RanBPM [46,47] (reviewed in [22,23]). Understanding the full spectrum of effects of the CTLH complex activity will require a better characterization of the activity of the complex and the identification of its targets.

## 4. Materials and Methods

### 4.1. Plasmid Constructs

pCMV-HA-RanBPM and deletion mutants have been previously described [34,36]. pEBG-GST-ΔN-c-Raf was a gift from Dr. Zhijun Luo (Boston University, Boston, MA, USA). pET28a-ΔN-c-Raf was generated by isolating ΔN-c-Raf from pEBG-GST-ΔN-c-Raf and subcloning into pET28a (EMD Millipore, Billerica, MA, USA). pBSK-HA-Ubiquitin was a gift from Dr. Lina Dagnino (Western University, London, ON, Canada). pCGN-HA-RMND5A was obtained by subcloning RMND5A cDNA obtained by RT-PCR from a Jurkat T cell cDNA library into pCGN-HA plasmid. All pGEX4T1-GST-RanBPM wild-type (WT) and mutant constructs were generated by polymerase chain reaction (PCR) and subcloned in the pGEX4T1 vector (GE Healthcare Life Sciences, Little Chalfont, UK). RanBPM point mutants (pGEX-4T-1-GST-C4 Q703L T750L and pCMV-HA-RanBPM R625L E626L) were generated by site-directed mutagenesis. All PCR reactions were performed using KOD Hot Start Polymerase PCR kit (EMD Millipore).

### 4.2. Cell Lines and Cell Culture and Transfections

HeLa and HEK293 cells were obtained from the American Type Culture Collection (Manassus, VA, USA). Cells were grown in high-glucose DMEM supplemented with 10% FBS, 1% sodium pyruvate, 1% l-glutamine, and 4.5 g/L glucose at 37 °C and 5% CO_2_. HeLa cell lines stably expressing either RanBPM shRNA (clone 2–7) or control shRNA were previously described [34]. To generate HeLa and HEK293 RMND5A CRISPR/Cas9 knockout cells, guide RNA targeting exon 3 of RMND5A (top oligo: 5′-CACCGTGGAGCACTTCTTTCGACA-3′; bottom oligo: 5′-AAACTGTCGAAAGAAGTGCTCCAC-3′) were cloned into pSpCas9(BB)-2A-Puro V2.0 (PX459). Forty-eight hours after transfection, cells were put under puromycin selection (1.2 μg/mL for HEK293, 0.3 μg/mL for HeLa) for seven days, followed by colony picking and expansion. The targeted region and the top predicted off-target region were checked by sequencing for clone #1 which was used throughout this study (Figure S4).

Mouse embryonic fibroblasts were prepared from E13.5 embryos according to standard protocols. HEK293 cell growth rates were assessed as previously described [21] with WT HEK293 and RMND5A KO clones #1, 3 and 14 (see Figure S3). A similar procedure was employed to evaluate MEFs growth rates, except that cells were kept in serum-containing media.

For transfection experiments, ExGen 500 in vitro Transfection Reagent (Fermentas Thermo Fisher Scientific Inc., Waltham, MA, USA), TurboFect Transfection Reagent (Thermo Fisher Scientific Inc., Waltham, MA, USA) and JetPRIME Transfection Reagent (PolyPlus Transfection, Illkirch, France) were all used according to the manufacturer’s instructions. For each pCMV-HA RanBPM deletion mutant, the amount of construct transfected was adjusted to account for variations in stability between the expressed proteins. Transfected cells were incubated 24–48 h at 37 °C in 5% carbon dioxide (CO_2_).

### 4.3. In Situ Proximity Ligation Assay (PLA)

Duolink II (Sigma-Aldrich Inc., St. Louis, MO, USA) in situ proximity ligation assay (PLA) was performed as previously described [48]. Cover slips were mounted onto glass slides with Prolong Gold antifade reagent with DAPI (Molecular Probes by Life Technologies, Burlington, ON, Canada) and were analyzed with an Olympus BX51 microscope (Olympus America Inc., Center Valley, PA, USA). Images were captured using Image-Pro Plus v4.5 software (Media Cybernetics Inc., Bethesda, MD, USA). The primary antibodies used were: c-Raf (1:50, E-10, Santa Cruz, Santa Cruz, CA, USA), Hsp90 (1:100, H-114, Santa Cruz), RanBPM (1:400, K-12, Santa Cruz), and MAEA (1:200, ab65239, Abcam, Cambridge, UK).

### 4.4. Cell Extracts and Western Blot Analyses

For binding experiments, whole cell extracts (WCE) were prepared as described [21,34] and for Western blot analyses, extracts were prepared in radioimmunoprecipitation assay (RIPA) buffer (50 mM Tris (pH 8.0), 150 mM NaCl, 0.1% SDS 0.5% sodium deoxycholate, 1% Triton X-100, 1 mM EDTA) supplemented with 10 μg/mL aprotinin, 2 μg/mL leupeptin, 2.5 μg/mL pepstatin, 1 mM DTT, 2 mM NaF, 2 mM Na3VO4, and 0.1 mM PMSF. The cell lysates were centrifuged at 4 °C for 20 min at 13,000 rpm.

Samples were resolved by sodium dodecyl sulfate–polyacrylamide gel electrophoresis (SDS-PAGE) on either 8% or 10% acrylamide gels and transferred onto a polyvinylidene fluoride (PVDF) membrane, blocked in 5% non-fat dry milk in Tris Buffered Saline with Tween 20 (TBS-T) or in Odyssey Blocking Buffer (Li-COR Biosciences, Lincoln, NE, USA), and hybridized with the following antibodies: c-Raf (C-12, 1:500, Santa Cruz), HA (HA-7, 1:1000, Sigma–Aldrich, St. Louis, MO, USA), RMND5A (NBP1–92337, 1:300, Novus Biologicals, Littleton, CO, USA), β-actin (I-19, 1:2000, Santa Cruz), RanBPM (5M, 1:2000, Bioacademia, Osaka, Japan), GST (B-14, 1:500, Santa Cruz), A-Raf (1:500, Cell Signaling Technology, Danvers, MA, USA, #4432), B-Raf (1:1000, Cell Signaling Technology #9434), phospho-T202/Y204-ERK1/2 (1:2000, Cell Signaling #4370), ERK1/2 (1:1000, Cell Signaling #9102), phospho-S217/221-MEK1/2 (1:2000, Cell Signaling #9154), MEK1/2 (1:1000, Cell Signaling #9122). Following antibody incubations, blots were developed using either Western Lightning Enhanced Chemiluminescence (ECL) Substrate (Perkin Elmer Inc., Waltham, MA, USA) or Clarity Western ECL Substrate (Bio-Rad Laboratories Inc., Hercules, CA, USA). Images were captured using either Kodak X-OMAR LS film (Carestream Health Inc., Rochester, NY, USA) or a ChemiDoc MP (Bio-Rad Laboratories Inc.) and Image Lab software (Bio-Rad Laboratories Inc.).

### 4.5. GST Pull-Down Assays

For pull-down assays using Hela cell extracts, extracts were brought up to a final concentration of 0.4% NP40, 0.4% Triton X-100, 20 μg/mL aprotinin, 4 μg/mL leupeptin, 5 μg/mL pepstatin, 2 mM DTT, 4 mM NaF, 4 mM NaVO4, and 0.2 mM PMSF and incubated with Glutathione-Agarose beads (Sigma-Aldrich Inc., St. Louis, MO, USA) overnight at 4 °C. Beads were subsequently washed three times in WCE buffer supplemented with 0.4% NP40, 0.4% Triton X-100, 1 mM DTT and 0.1 mM PMSF, resuspended and boiled in SDS loading dye and the resulting supernatant was collected and analyzed by Western blot.

For pull-down assays using *E. coli* extracts, bacterial expression constructs were transformed into *E. coli* strain BL21DE3. Protein expression was induced with 0.1 mM IPTG and extracts prepared lysed in GST protein lysis buffer (25 mM HEPES pH 7.4, 100 mM KCl, 2 mM EDTA, and 20% glycerol). Pull-downs were performed in binding buffer (15 mM HEPES pH 7.4, 60 mM KCl, 2 mM EDTA and 12% glycerol, 0.6% NP40, 0.6% Triton-X) for 2 h at 4 °C with Glutathione-Agarose beads. Each sample was then incubated with ΔN-c-Raf extracts for 2 h at 4 °C and beads were washed with binding buffer and analyzed by Western blot.

For analyzing c-Raf ubiquitination, cells were co-transfected with pEBG-GST-ΔN-c-Raf and pBSK-HA-Ubiquitin. Twenty-four hours after transfection, cells were incubated with 10 μM MG132 (EMD-CalBiochem, San Diego, CA, USA) for 8 h. Cells were lysed in denaturing buffer (50 mM Tris, pH 7.5, 150 mM NaCl, 1% Triton, 1% SDS, 1 mM Na3VO4, 10 mM NaF, and 5 mM NEM (N-Ethylamalide, Bioshop Canada, Burlington, ON, Canada)), incubated on ice for 30 min, boiled for 10 min, and then back on ice for 1 h. Lysates were diluted 1:5 in buffer A (50 mM Tris, pH 7.5, 150 mM NaCl, 1% Triton, 1 mM Na3VO4, 10 mM NaF, and 5 mM NEM) and incubated with Glutathione-Agarose beads for 2 h at 4 °C. Beads were then washed three times in buffer A, resuspended and boiled in SDS loading dye, and analyzed by Western blot.

### 4.6. Mouse Tumour Models

Experiments were performed in accordance with the Canadian Council on Animal Care (CCAC) guidelines approved by the Western University Animal Care Committee (AUP# 2017-140, 2/01/2018). For subcutaneous injections, 500,000 cells in 100 µL of RPMI with Matrigel (1:1) were injected into the right flank of 6–8 week old NOD/SCID/gamma (NOD.Cg-Prkdcscid Il2rgtm1Wjl/SzJ, NSG) mice (The Jackson Laboratory). Tumour measurements were taken twice per week and a digital caliper was used to measure Length × Width × Depth of the tumour upon excision in order to calculate volume. Mice were fed regular chow of chow containing Doxocycline (0.625 g doxycyline hyclate/kg; TestDiet, Richmond, IN, USA).

### 4.7. Statistical Analyses

One-way analysis of variance (ANOVA) with post-hoc Tukey test was performed to compare multiple groups and two-sample *t*-test assuming unequal variance was performed to compare pairs of groups. Graphed data are presented as mean ± standard error of the mean (SEM) or standard deviation (SD), as indicated in figure legends and are determined to be significant when *p* < 0.05.

## 5. Conclusions

Our study reveals that Raf stability is regulated by the CTLH complex, a still poorly characterized E3 ubiquitin ligase complex. We show that the CTLH complex subunit RanBPM directly binds to c-Raf through its C-terminal CRA domain and that c-Raf ubiquitination and degradation is promoted through the CTLH complex. We also provided evidence that this regulation may pertain to the related kinases A- and B-Raf, suggesting a common mechanism of regulation of all three Raf kinases by the CTLH complex. Finally, we show that loss of CTLH function through knockout of CTLH subunits RMND5A and RanBPM promotes cell proliferation and tumour formation, suggesting that the CTLH complex may restrict tumorigenesis at least in part through inhibition of the ERK pathway through a control of c-Raf expression levels.

## Supplementary Materials

Supplementary materials can be found at https://www.mdpi.com/1422-0067/20/4/934/s1.

## Abbreviations

CHIP	C-terminus of constitutive heat shock protein (Hsc) 70-interacting protein
CR3	Conserved Region 3
CRA	CT11-RanBPM
CTLH	C-terminal to LisH (LIS1-homology motif)
ELF3	E74-like factor 3
ERK	Extracellular signal-Regulated Kinase
Hsp90	Heat Shock Protein 90
KO	Knockout
L1CAM	L1 cell adhesion molecule
MAEA	Macrophage erythroblast attacher
RanBPM	Ran Binding Protein M
RMND5A	Required for Meiotic Nuclear Division 5A
RON	Recepteur d’origine nantais
TG2	transglutaminase 2
XIAP	X-linked inhibitor of apoptosis protein
WT	Wild-type
