# Supplementary material for: Regulation of c-Raf Stability through the CTLH Complex
Source: Int J Mol Sci. 2019 Feb 21;20(4):934. doi: 10.3390/ijms20040934 (PMC6412545; doi:10.3390/ijms20040934)
Supplement: Supplementary file 1 [file ijms-20-00934-s001.pdf]

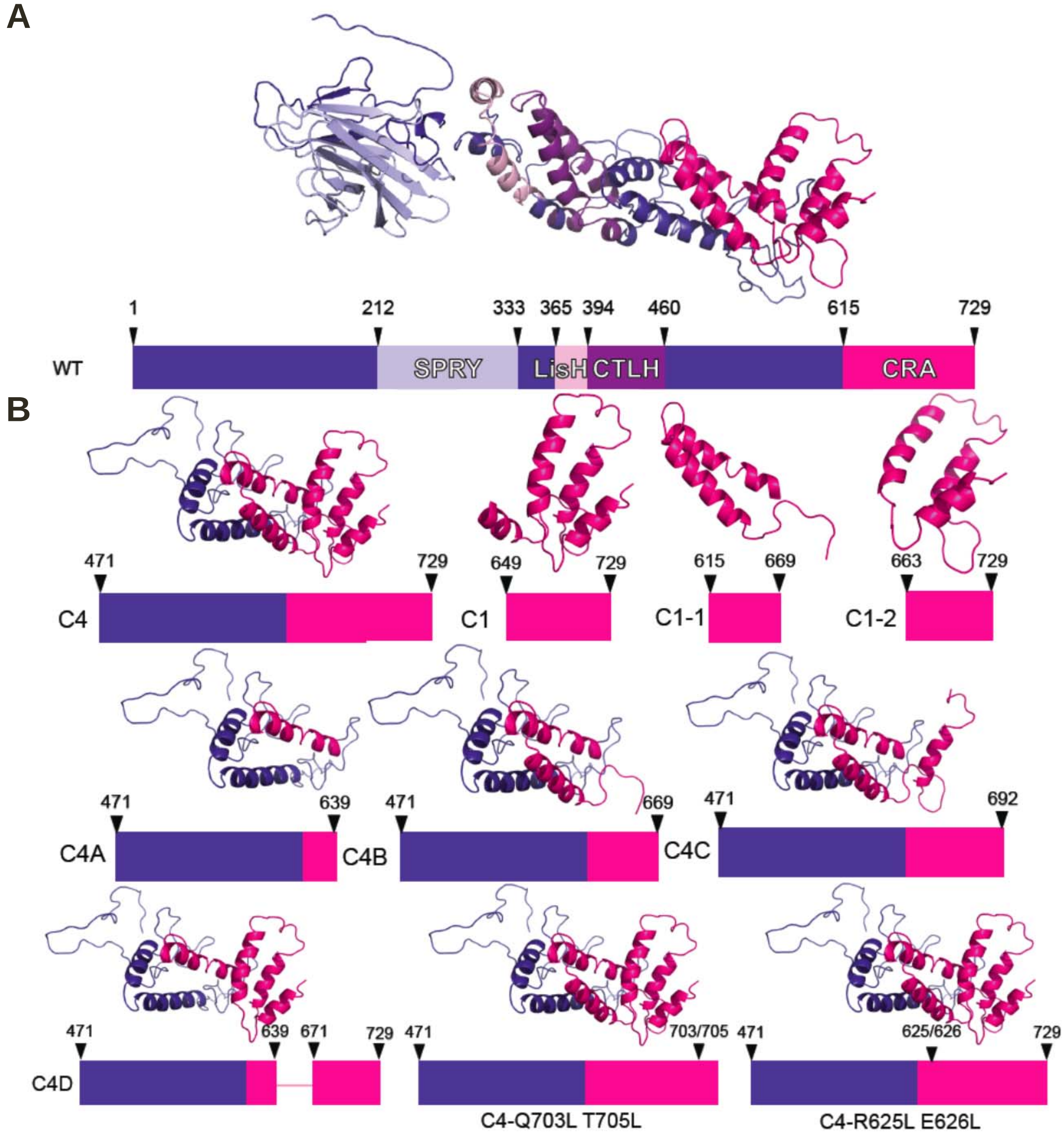

Supplementary Figure 1 Schematics and predicted structures of RanBPM and the C-terminal CRA domain mutants of RanBPM. **A.** Schematics of RanBPM with the domain indicated and predicted stucture shown above. **B.** Schematics of the RanBPM mutants subcloned in the pGEX-4T-1 vector with the predicted sructure shown above.

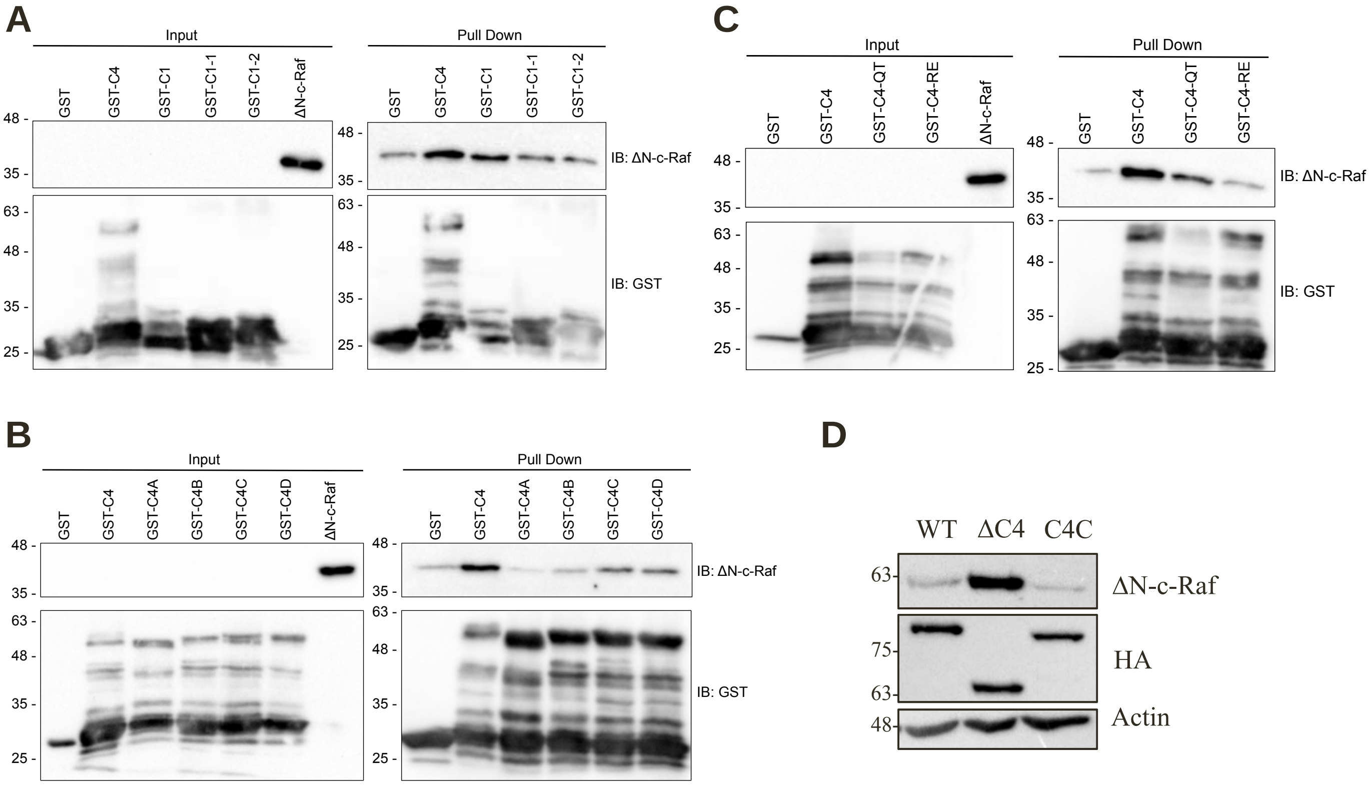

Supplementary Figure 2. The CRA domain of RanBPM interacts directly with ΔN-c-Raf. GST pull-down assays were performed using GST, GST-C4, GST-C1, GST-C1-1 and GST-C1-2 (**A**); GST-C4A, GST-C4B, GST-C4C, and GST-C4D (**B**); and GST-C4-Q703L/T705L, and GST-C4-R625L/E626L (**C**) *E. coli* extracts as well as ΔN-c-Raf *E. coli* extracts. Pull-downs were analyzed by Western blot with C-Raf and GST antibodies to detect ΔN-c-Raf and GST-fusion proteins, respectively. **D**. Analysis of RanBPM C4C mutant in mammalian cells. HeLa RanBPM shRNA cells were transfected with pEBG-GST-ΔN-c-Raf and either pCMV-HA RanBPM, RanBPM-ΔC4, or RanBPM-ΔC4C, and whole cell extracts were prepared 24 h post-transfection and analyzed by Western blot. HA, c-Raf and β-actin antibodies were used to detect HA-RanBPM constructs, ΔN-c-Raf and β-actin proteins, respectively.

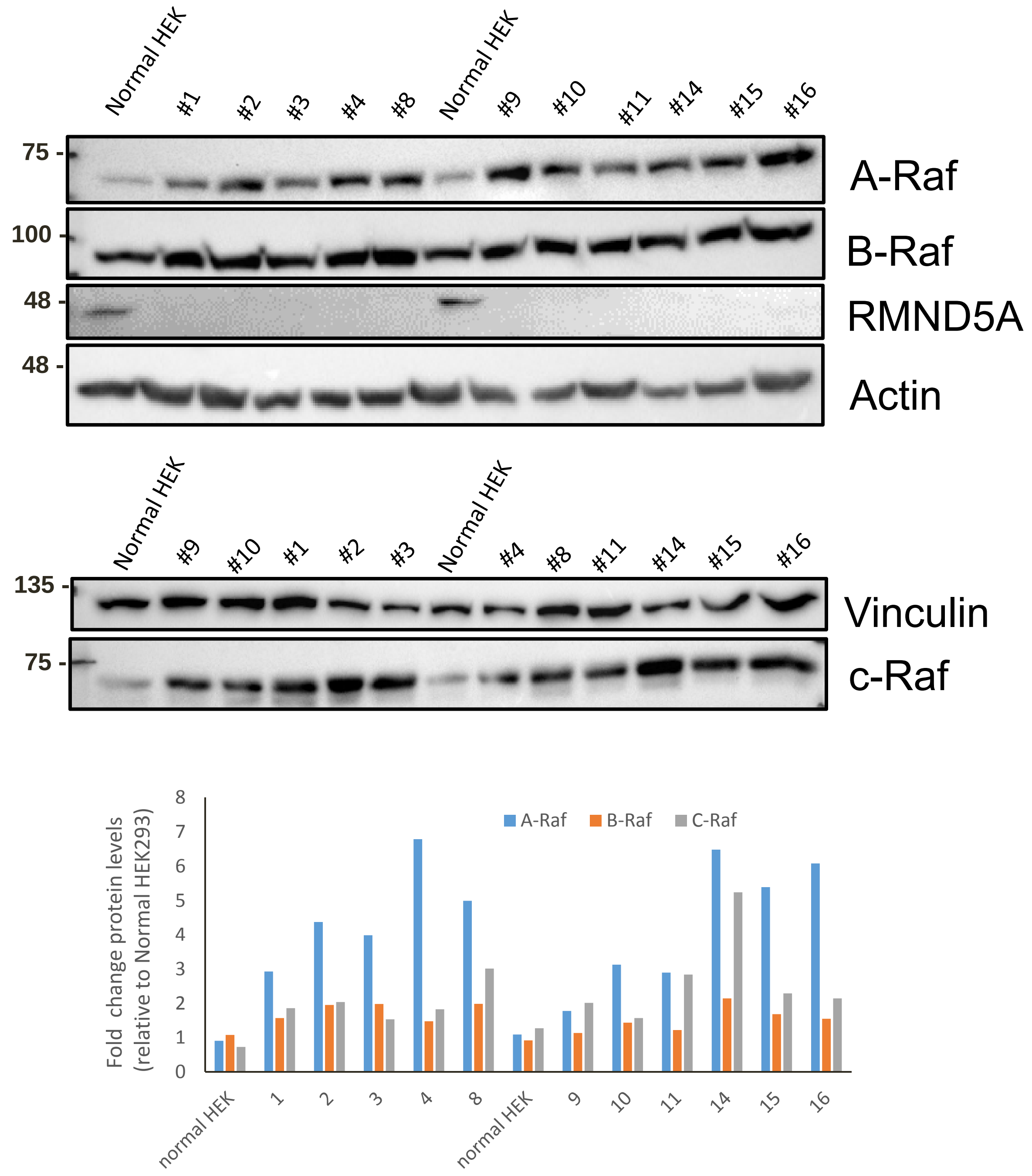

Supplementary Figure 3. Western Blot analysis of WT HEK293 or RMND5 KO clonal derivatives. Blots were hybridized as indicated in the figure and materials and methods. Same extracts were analyzed on two separate blots as shown. Quantification of band intestity normalized to Actin (a-Raf, b-Raf) or vinculin (c-Raf) is shown below.

Supplementary Figure 4

Analysis of on-target and off-target sites of RMND5A HEK293 KO clone #1

Guide design by benchling.com

Guide sequence: 5'GTGGAGCACTTCTTTCGACA PAM: AGG

On-target Score = 65.7 Off-target score = 86.0

Targets exon 3 of RMND5A (guide sequence shaded)

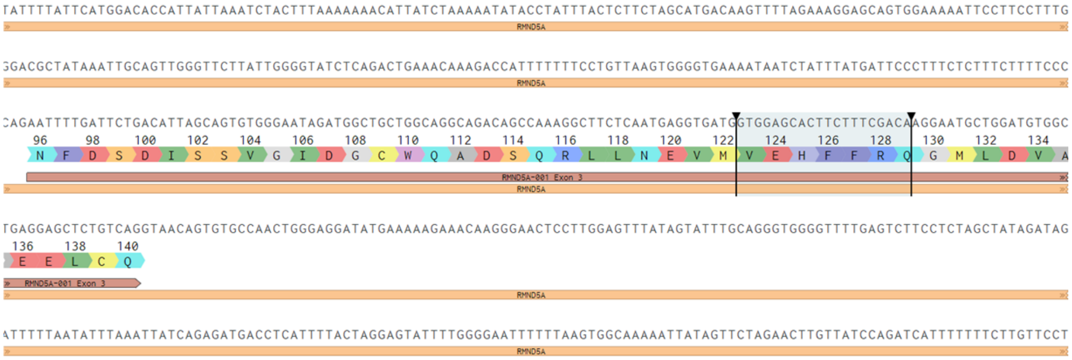

Analysis of Indels in RMND5A KO cells (guide sequence underlined):

On-target

Reverse sequencing of control cells:

NNNNNNNNNNNNCTGGNNACAGTTCTAGACTATAATTTTTGCCACTTAAAAAATTCCCCAAAATACTCCTAGTAAA
ATGAGGTCATCTCTGATAATTTAAATATTAAAAATCTATCTATAGCTAGAGGAAGACTCAAAACCCCACCCTGCAA
ATACTATAAACTCCAAGGAGTTCCCTTGTTTCTTTTTCATATCCTCCCAGTTGGCACACTGTTACCTGACAGAGCT
CCTCAGCCACATCCAGCATTCCTTGTCNAAAGAAGTGCTCCACCATCACCTCATTGAGAAGCCTTTGGCTGTCTG
CCTGCCANCANCCATCTATTCCCACACTGCTAATGTCANAATCAAAATTCTGGGGAAAAGAAAGAGAAAGGGAAT
CATAAATAGATTATTTTCACCCCACTTAACAGGAAAAAAATGGTCTTTGTTTCAATCTGANATACCCCAATAAGAAC
CCAACTGCAATTTATANCGTCCCAAAGGAAGGAATTTTTCCACTGCTCCTTTCTAAAACTTGTCATGCTANAANAN
TAAATANGTATATTTTTAGATAATGTTTTTTTAAAGTAGATTTAATAATGGTGTCCATGAATAAAATAAAATCAAGCN
AATGAACTGATTTCTANGNNTTATATGCANCAATATTTTTAGAGGAATGNNNTGANNANNNANNAAANCATGCNC
ACANNANNNNAAN

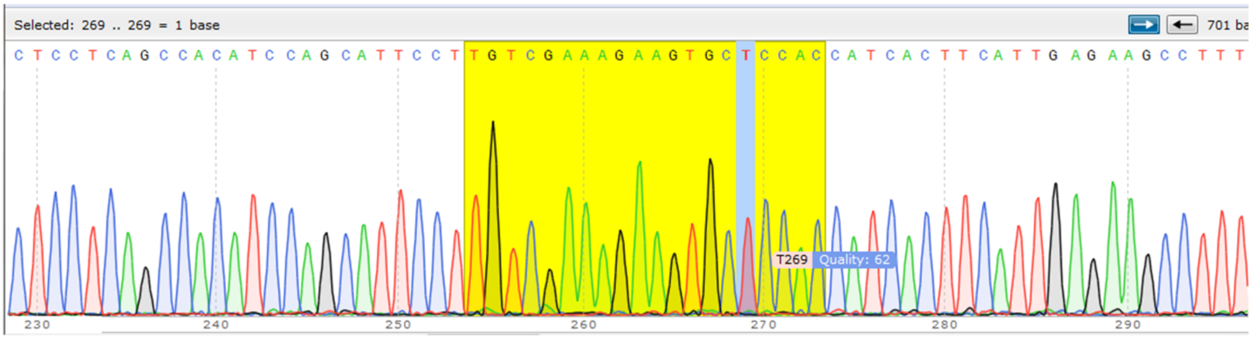

Reverse sequencing of RMND5A KO cells:

NNNNNNNNNNNNNNCTGGNNANNNTTCTAGAACTATAATTTTTGCCACTTAAAAAATTCCCCAAAATACTCCTAGTA
AAATGAGGTCATCTCTGATAATTTAAATATTAAAAATCTATCTATAGCTAGAGGAAGACTCAAAACCCCACCCTGC
AAATACTATAAACTCCAAGGAGTTCCCTTGTTTCTTTTTCATATCCTCCCAGTTGGCACACTGTTACCTGACAGAG
CTCCTCAGCCACATCCAGCATTCCTTGTCCNAANNANNNGNNCCNCCTTCNCTTCNTTGANAANNCNTNNNCTG
NANGNNAGCNNGCNNCCATCTATTCCCACNCTGCTAATGTCNGAATCNNAATTCNGGGGAAAANAANNANAANG
GNAATCATAANTANATTATTTTCACCCCGCTTANCNNGAAAAAAATGGNCTTTGTTTCAGTCTGANATACCCCTNT
ANNANCCCNACTGCNNNTTATANCGTACCAAAGGANGGAATTTTTCCNCTGNTCCTTTCNAAAANTTGNNNTGNT
NNAANANTAAATANGTNTATTTNNNNNTAANGNTTTTTTAAAGTANATTTAATAATGGNGTNNNTNANNNNNNNNN
NNNCAANNNNNNNNNCGGNTTTCTAGGNATTANATGCANNNNTATTTTTAGAGNANTGNAATGNNNNNNNNNNN
NNGNNTNNNGNNNANNANNANAANNNNCGAATGAACTGATTTCTAGGCATTATATGCANCAATATTTTTAGAGGA
NTGCAATGATGACNTGNNNNNNNNNNNNNNNNNNNNNNNNNNNNNNNNNNNNNNNNNNNNNNNNNNNNNNNNN
NNNNNNNNNNNNNNNN

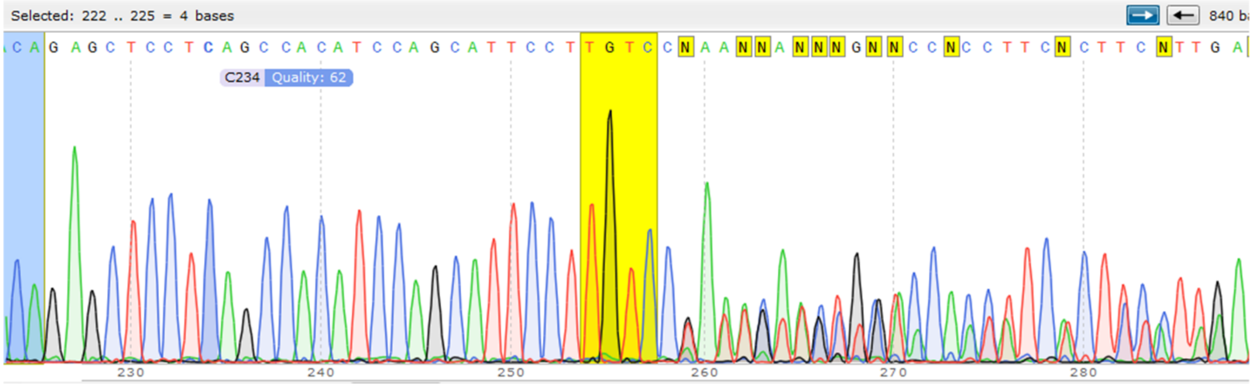

Off-target

Sequence	PAM	Score	Gene	Chromosome	Strand	Position	Mismatches	On-target
GTGGAGCACTTCTTTCGACA	AGG	100	ENSG00000153561	chr2	1	86979117	0	TRUE
CAGAAGCATTTCTTTCGACA	TAG	0.934201		chr8	-1	1.35E+08	4	FALSE
GTGGGGGACTTCTTTCTACA	GGG	0.881154		chr19	-1	53219258	3	FALSE
GTGGGGGACTTCTTTCTACA	GGG	0.881154		chr19	1	53046429	3	FALSE
CTGGAGCACTGCTTTCAACA	CAG	0.861959		chr10	1	1.06E+08	3	FALSE

Analysis of top predicted off target site:

Forward sequencing of control cells:

NNNNNNNNNNNNNNANNNNNNCCCCTTGACTGCATTCTTTAGAAATAGAGGAAAGATTGTATCCCTTGATGATGTC
TGTCAGCTTGAGAGAGCTATGAATCACAGCAAAATGGCATTTTATATTTAGTTATTCATTTCCCATTCAAATCCAG
GTGACAAACCTTGTGCAAAACAGCTCCGTATGACTCACATCAACTTTTAAAGATGTTGGTAACGAAGTCTGACGA
ATCGTTATTGACCCAGCTTTGAGAGTGTACACAGTTTATCTCAGCTATTAATAAGATATGCAATCTTCATCTGGTT
CAGAACATAATTATTATTATTTTCTCCCTCGTCATGACTCTCATGACAAGAGGGAAGATGTATTCCTGACTTAACA
GAGCGTCTGTGAGCTATGTCAAAAGAAATGCTTCTGGATTTTACCACATCTAGAAGTCTTGGAATGGATTCTAGA
TATAAGACAAAAGTGAGGAACACATAAAAATGGGCATGTTACAATAATAATAATTTTATTATTAATAGATCAATAAT
TTATTAATGTTAAAATTTATATAATAAAAAGTGCAACAGGTTGAAGAGATGCTAAACATACAGGTGGTTCAGAATC
ACCCCATTGTTGTTTCTTGTGGCTCAAGCCTGGGTTTTTTTCTATCATCCCCTCTGATTGGTTGTGCCAAATCTGA
CCTTTGTTATTTAATTAGTTAACTCATTTTTTGAATGGATAGGTATTCAGAAAACATATNANNNTGCTGGAATATGA
AGCTGGNATNAAGGNNNN

Forward sequencing of KO cells:

NNNNNNNNNNNNNNNNNNNNNTGNNNCTTGACTGCATTCTTTAGAAATAGAGGAAAGATTGTATCCCTTGATGATGTCTGTCAGCTTGAG
AGAGCTATGAATCACAGCAAAATGGCATTTTATATTTAGTTATTCATTTCCCATTCAAATCCAGGTGACAAATCTTGTGCAAAACAGCTCCGTA
TGACTCACATCAACTTTTAAAGATGTTGGTAACGAAGTCTGACGAATCGTTATTGACCCAGCTTTGAGAGTGTACACAGTTTATCTCAGCTATT
AATAAGATATGCAATCTTCATCTGGTTCAGAACATAATTATTATTATTTTCTCCCTCGTCATGACTCTCATGACAAGAGGGAAGATGTATTCCTG
ACTTAACAGAGCGTCTGTGAGCTATGTCAAAAGAAATGCTTCTGGATTTTACCACATCTAGAAGTCTTGGAATGGATTCTAGATATAAGACAA
AAGTGAGGAACACATAAAAATGGGCATGTTACAATAATAATAATTTTATTATTAATAGATCAATAATTTATTAATGTTAAAATTTATATAATAA
AAAGTGCAACAGGTTGAAGAGATGCTAAACATACAGGTGGTTCAGAATCACCCCATTGTTGTTTCTTGTGGCTCAAGCCTGGGTTTTTTTCTAT
CATCCCCTCTGATTGGTTGTGCCAAATCTGACCTTTGTTATTTAATTAGTTAACTCATTTTTTGAATGGATAGGTATTCAGAAAACATNTNANGT
GCTGGAATATGAAGCTGGATAAGGCNNNNNNNNNNNNNNNNNNTGCCTTNNNCANNNNNNNNNNNNNNTNGTNNNNNNNNNNNNNN
NNGNNNNNNNTGNNNNNANTCT

NCBI blast search control cells (query) versus KO cells (subject):

```
Download  Graphics

Sequence ID: Query_59419  Length: 856  Number of Matches: 1

Range 1: 27 to 767 Graphics                         Next Match  Previous Match

Score            Expect   Identities       Gaps          Strand
1312 bits(1454)  0.0      738/742(99%)     1/742(0%)     Plus/Plus

Query  24   CTTGACTGCATTCTTTAGAAATAGAGGAAAGATTGTATCCCTTGATGATGTCTGTCAGCT  83
            ||||||||||||||||||||||||||||||||||||||||||||||||||||||||||||
Sbjct  27   CTTGACTGCATTCTTTAGAAATAGAGGAAAGATTGTATCCCTTGATGATGTCTGTCAGCT  86

Query  84   TGAGAGAGCTATGAATCACAGCAAAATGGCATTTTATATTTAGTTATTCATTTCCCATTC  143
            ||||||||||||||||||||||||||||||||||||||||||||||||||||||||||||
Sbjct  87   TGAGAGAGCTATGAATCACAGCAAAATGGCATTTTATATTTAGTTATTCATTTCCCATTC  146

Query  144  AAATCCAGGTGACAAACCTTGTGCAAAACAGCTCCGTATGACTCACATCAACTTTTAAAG  203
            |||||||||||||||| |||||||||||||||||||||||||||||||||||||||||||
Sbjct  147  AAATCCAGGTGACAAATCTTGTGCAAAACAGCTCCGTATGACTCACATCAACTTTTAAAG  206

Query  204  ATGTTGGTAACGAAGTCTGACGAATCGTTATTGACCCAGCTTTGAGAGTGTACACAGTTT  263
            ||||||||||||||||||||||||||||||||||||||||||||||||||||||||||||
Sbjct  207  ATGTTGGTAACGAAGTCTGACGAATCGTTATTGACCCAGCTTTGAGAGTGTACACAGTTT  266

Query  264  ATCTCAGCTATTAATAAGATATGCAATCTTCATCTGGTTCAGAACATAATTATTATTATT  323
            ||||||||||||||||||||||||||||||||||||||||||||||||||||||||||||
Sbjct  267  ATCTCAGCTATTAATAAGATATGCAATCTTCATCTGGTTCAGAACATAATTATTATTATT  326

Query  324  TTCTCCCTCGTCATGACTCTCATGACAAGAGGGAAGATGTATTCCTGACTTAACAGAGCG  383
            ||||||||||||||||||||||||||||||||||||||||||||||||||||||||||||
Sbjct  327  TTCTCCCTCGTCATGACTCTCATGACAAGAGGGAAGATGTATTCCTGACTTAACAGAGCG  386

Query  384  TCTGTGAGCTATGTCAAAAGAAATGCTTCTGGATTTTACCACATCTAGAAGTCTTGGAAT  443
            ||||||||||||||||||||||||||||||||||||||||||||||||||||||||||||
Sbjct  387  TCTGTGAGCTATGTCAAAAGAAATGCTTCTGGATTTTACCACATCTAGAAGTCTTGGAAT  446

Query  444  GGATTCTAGATATAAGACAAAAGTGAGGAACACATAAAAATGGGCATGTTACaataataa  503
            ||||||||||||||||||||||||||||||||||||||||||||||||||||||||||||
Sbjct  447  GGATTCTAGATATAAGACAAAAGTGAGGAACACATAAAAATGGGCATGTTACAATAATAA  506

Query  504  taattttattattaatagatcaataatttattaatgttaaaatttatataataaaaaGTG  563
            ||||||||||||||||||||||||||||||||||||||||||||||||||||||||||||
Sbjct  507  TAATTTTATTATTAATAGATCAATAATTTATTAATGTTAAAATTTATATAATAAAAAGTG  566

Query  564  CAACAGGTTGAAGAGATGCTAAACATACAGGTGGTTCAGAATCACCCCATTGTTGTTTCT  623
            ||||||||||||||||||||||||||||||||||||||||||||||||||||||||||||
Sbjct  567  CAACAGGTTGAAGAGATGCTAAACATACAGGTGGTTCAGAATCACCCCATTGTTGTTTCT  626

Query  624  TGTGGCTCAAGCCTGGGtttttttCTATCATCCCCTCTGATTGGTTGTGCCAAATCTGAC  683
            ||||||||||||||||||||||||||||||||||||||||||||||||||||||||||||
Sbjct  627  TGTGGCTCAAGCCTGGGTTTTTTTCTATCATCCCCTCTGATTGGTTGTGCCAAATCTGAC  686

Query  684  CTTTGTTATTTAATTAGTTAACTCATTTTTTGAATGGATAGGTATTCAGAAAACATATNA  743
            |||||||||||||||||||||||||||||||||||||||||||||||||||||||| |||
Sbjct  687  CTTTGTTATTTAATTAGTTAACTCATTTTTTGAATGGATAGGTATTCAGAAAACATNTNA  746

Query  744  NNNTGCTGGAATATGAAGCTGG  765
             | |||||||||||||||||||
Sbjct  747  -NGTGCTGGAATATGAAGCTGG  767
```

Note: base 160 in control cells may potentially be a C, see trace:
